# Enhancing the anti-ageing, antimicrobial activity and mechanical properties of surface-coated paper by Ag@TiO_2_-modified nanopigments

**DOI:** 10.1007/s11356-022-20935-2

**Published:** 2022-05-24

**Authors:** Marwa Samir, Ramadan A. Geioushy, Samya El-Sherbiny, Osama A. Fouad

**Affiliations:** 1 Paper and Printing Laboratory, Chemistry Department, Faculty of Science, Helwan University, Helwan, Egypt; 2 Nanostructured Materials and Nanotechnology Department, Advanced Materials Institute, Central Metallurgical R & D Institute (CMRDI), P.O. Box, 87, Helwan, 11421 Cairo Egypt

**Keywords:** Ag@TiO_2_, Coated paper, Mechanical properties, Optical properties, Antimicrobial activity, Paper ageing

## Abstract

In this work, the effect of using Ag-doped TiO_2_ nanopigments on optical, mechanical and antimicrobial properties of coated paper was explored. Furthermore, the long-term antimicrobial activity of the coated paper was examined for up to 25 years. Titanium dioxide (TiO_2_) nanoparticles have been synthesized and doped with different percentages of Ag nanoparticles (Ag NPs) using a simple wet chemical approach. The Ag@TiO_2_ modified nanopigments were in the form of nanorods with an average size of about 20 nm as observed from TEM images. Increasing Ag content from 0.01 to 1.0% showed an increase in the mechanical properties of coated paper in terms of tensile, stretching, tensile energy absorption and burst while preserving the optical properties. Moreover, the antimicrobial inhibition activity increased with increasing the Ag content. The 1% Ag@TiO_2_ showed a long-lasting antimicrobial effect against *Staphylococcus aureus* (*S. aureus*) Gram-positive bacteria even after 25 years of ageing (93.4% inhibition). Investigation of reactive oxygen species (ROS) generation and reaction mechanism of antimicrobial activity over Ag/TiO_2_ under visible light is proposed. These results suggest that Ag/TiO_2_ NPs can be potentially used as a disinfection coating for paper and improving its mechanical properties.

**Graphical abstract:**

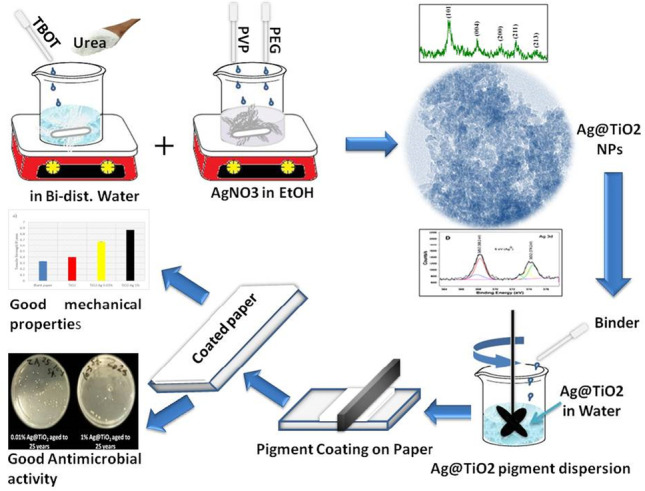

## Introduction

Paper is a primary medium for communication, education, artistic products, packaging and hygienic and industrial applications. It is for a long time being the method of communication, messages and transfer of knowledge along in different eras and civilizations. In addition, for now, it is being the most predominant tool for international documentation and large currency exchange. However, paper production finds itself facing new challenges with intense competition in rising raw materials, energy costs and the increase of bacterial and viral infections, including the recent coronavirus outbreak (Ezeudu et al. 2019; Hasan et al. 2020; Liu et al. 2016; Smith 2011). Paper easily suffers from biological attacks and degradation due to its inherent non-antimicrobial activity. Several microbial containments are viable even in libraries and archives; the extent of their effectiveness depends on the environmental situations and the composition of the substrate. Insects and moulds are the most common reasons for biological problems (Area and Ceradame 2011).

Surface coating is a simple and convenient way to introduce organic and/or inorganic antimicrobial compounds into cellulose paper, giving it antibacterial properties (Wu et al. 2018).

In paper coating, minerals are applied as white pigments to improve its visual appearance and its printability by filling cavities, covering fibres and smoothing paper surfaces. Furthermore, one of the most common paper coating objectives is to improve printing quality, image reproduction and surface strength (El-Sherif et al. 2017; Kasmani et al. 2013). All these criteria are achieved by various coating formulations, the right coating method and finishing treatments (Blechschmidt et al. 2013).

Pigment is the main constituent in the coating mixture, and it is responsible for controlling the coating components’ qualities (Gullichsen and Paulapuro 2000). Pigments arise from several minerals, like kaolin clay, calcium carbonate, silica and titanium dioxide pigment; these pigments can be used alone or mixed (Ahmed et al. 2012; El-Sherbiny et al. 2015; Morsy et al. 2014).

Metal oxide nanoparticles are considered to be one of the most interesting materials in a wide variety of applications due to their exceptional optical, electronic, photocatalytic and biological characteristics (Kannan et al. 2020). TiO_2_ nanoparticle has been employed as a highly competent white pigment in paper coating for its points of interest, such as high refractive index, excellent brightness, high level of whiteness, ideal hiding power, antimicrobial activity and insolubility in alkaline and acidic (El-Sherbiny et al. 2014; Morsy et al. 2016).

TiO_2_’s antimicrobial properties are related to the generation of reactive oxygen species (ROS) such as hydroxyl radicals in response to ultraviolet irradiation, a mechanism similar to TiO_2_’s photodegradation of organic contaminants. One of the main reasons for TiO_2_’s popularity is its ability to be synthesized in a variety of nanostructures, such as nanofibers, which addresses several inherent issues of particle agglomeration present in TiO_2_ nanoparticles and could be used in potential applications in sanitation and sterilization (Hashimoto et al. 2005; Soo et al. 2020).

However, pure TiO_2_ shows oxidation catalysis activities through excitation upon exposure to UV light which enhances the antimicrobial activity (Hashimoto et al. 2005) but limits its application in paper products.

One approach to improve the antimicrobial effectiveness of TiO_2_ is to incorporate dopants with equivalent antimicrobial properties such as silver. Silver is one of the most widely used dopants and extensively investigated for application in antimicrobial, catalysis, surface-enhanced Raman scattering detection, photographic plates, as well as conducting pastes in electronic devices (Fouad 2013). Moreover, recently, a prepared fish gelatin-chitosan/TiO_2_-Ag anti-bacterial composite film has been investigated for food packaging and studied against Gram-positive and Gram-negative bacteria (Lin et al. 2020). Its high intrinsic antimicrobial capabilities have attracted a lot of interest in recent decades. Silver nanoparticles have a smaller particle size and a greater specific surface area, which results in increased silver ion release and better particle interaction, resulting in improved antibacterial characteristics (Albert et al. 2015; Kedziora et al. 2018). Several techniques such as chemical, physical and biological have been used for synthesis of various nanoparticles. Moreover, metal nanoparticles including silver nanoparticles can be synthesized through different chemical and green chemistry (using biological resources) approaches. Biological resources such as plant resources have bioactive molecules including proteins, carbohydrates and organic acids which have been reported as reducing, capping and stabilizing agents for nanoparticle synthesis (Hassanisaadi et al. 2021; Duan et al. 2015). It also plays a major role in the world of medicine as a source of natural compounds which can be used as anti-microbial, anti-inflammatory and anti-cancer agents (Varijakzhan et al. 2020). The biosynthesis of the nanoparticles by bioresources including algae, fungi and bacteria as greener synthesis can be considered based on the availability and cautious control (Soltys et al. 2021; Ab Razak et al. 2021). In addition, synthesis of Ag@TiO_2_ nanocomposites has been vigorously investigated by various physicochemical methods such as sol–gel, thermal dissociation, chemical vapour deposition and electrochemical oxidation for various vital applications. To overcome some obstacles in these methods, nowadays, researchers focus on the plant extract–mediated synthesis of Ag@TiO_2_ nanocomposites for improved photocatalytic and biological activities. However, this green synthesis methodology in most cases requires more than one step and more time for reaction process to be completed (Bhardwaj and Singh 2021).

Therefore, applying a simple and facile chemical route can be a promising alternative for nanoparticle synthesis. In this regard, polyvinylpyrrolidone, polyethylene glycol and some others have been successfully used as capping, stabilizing and reducing agents for nanoparticle synthesis (Ahmed et al. 2010; Geioushy et al. 2021).

The current work aimed to prepare antimicrobial Ag@TiO_2_-modified nanopigments with a low concentration of Ag (up to 1.0 wt.%) using simple chemical feasible techniques to impart dual functions in paper coating. TiO_2_ was functioned as the primary pigment for the coating mixture and Ag nanoparticles (Ag NPs) as a dopant to enhance the antimicrobial properties of TiO_2_.

Focusing on the preparation of the modified pigment in the form of doped pigments was to avoid the inhibition of the antimicrobial activity of Ag NPs by oxidation and aggregation. In addition, we emphasized the presence of Ag NPs at a small amount as their dark grey colour are not favoured for paper products.

The prepared materials with different Ag ratios were tested towards their effect on antimicrobial performance towards Gram-positive, Gram-negative bacterial and fungi organisms. To the best of our knowledge, no prior studies have examined the influence of Ag@TiO_2_ modified nanopigments on the anti-ageing and long-term antimicrobial activity of the coated paper. So, we investigated the effect of ageing up to 25 years on the antimicrobial efficiency of the prepared pigments. The prepared coated paper was also characterized in terms of optical and mechanical properties.

## Experimental

### Materials

The following materials were utilized to make Ag@TiO_2_-modified nanopigments in this study: tetrabutyl orthotitanate (TBOT, C_16_H_36_O_4_Ti, 97%) purchased from Sigma-Aldrich Company, silver nitrate (AgNO_3_, 99%) purchased from Dop Organic Kimya Company and Urea (NH_2_·CO·NH_2_, 99%) and ethanol (EtOH, C_2_H_5_OH, 95%) purchased from Adwic Company. Polyethylene glycol (PEG, Mwt = 8000) was used as a reducing agent supplied from MP Biomedicals Company. Polyvinylpyrrolidone (PVP K 15, Mol wt = 10,000) was used as a stabilizing agent supplied from Fluka Company. The materials used for coated paper preparation were copolymer of Acronal S 360D (based on *n*-butyl acrylate, styrene and acrylonitrile) as a binder and polyvinyl alcohol (PVOH) as a thickener supplied by BASF Germany. Sodium hexametaphosphate (Na_2_P_6_O_4_ supplied by Fine Chemicals was used as a dispersing agent.

### *Preparation of Ag@TiO*_*2*_* modified nanopigments*

Firstly, for the preparation of TiO_2_ nanoparticle (solution A) 1 g of urea and 200 ml of bi-distilled water were introduced into a beaker, and the solution was agitated for 5 min. Fifty millilitres of tetra butyl orthotitanate was added dropwise, and the resulting solution was agitated for 30 min. This suspension was introduced to a water bath for 1 h at 90 °C. The suspended particles were then filtered through a BÜchner system and washed exceptionally well with ethanol and distilled water several times. The obtained TiO_2_ pigment was then dried at 80 °C for 12 h.

Secondly, Ag@TiO_2_-modified nanopigments with two Ag concentrations of 0.01% and 1% were prepared by introducing the calculated amounts of silver nitrate into two separate TiO_2_ samples. AgNO_3_ was introduced into a beaker containing 50 ml (0.01mMol) polyethylene glycol and 50 ml (0.01mMole) polyvinylpyrrolidone (PVP) then the solution was stirred for 10 min (solution B). This solution was added dropwise into solution A (mentioned before). Then, the mixture solutions were stirred with 10 ml ethanol until the dissolution of silver salt and heated to 80 °C for about 2 h. The suspended particles were centrifuged and rinsed many times with distilled water and then with ethanol. These pastes were then dried at 80 °C for 12 h.

### Characterization

The prepared nanopigments were characterized physiochemically using the following techniques and methods: The purity formed phases, and crystallite sizes of Ag@TiO_2_-modified nanopigments were studied by X-ray powder diffraction (D/Max 2500 PC, Rigaku, Japan) using Cu target operating at 40 kV and 100 mA (with a scan speed of 4°/min) and Cu-Kα radiation of wavelength equals 1.54059 Å. The diffraction angle 2θ was scanned in the range 10°–80°. The morphology of the particles was characterized by a transmission electron microscope (TEM, JEM-2100, Japan) operated at 200 kV. Samples for TEM investigation were prepared by placing a drop of sonicated colloidal dispersion on the carbon-coated copper grid and then allowing the drops to dry in the air. The valence state and elemental composition of the prepared samples was characterized using a Thermo Scientific K-Alpha X-ray photoelectron spectroscopy (XPS) operated with an Al-Kα micro-focused monochromator within an energy range up to 4 keV. UV–Vis spectrum was recorded at room temperature using a spectrophotometer (UV–Vis, JASCO *V-570*, Japan). Photoluminescence (PL) spectra are collected at excitation wavelength of 320 nm using a luminescence spectrometer (Varian-CARY Fluorospectrophotometer).

### Antimicrobial activity

The antimicrobial activity of Ag@TiO_2_ modified nanopigments was assessed by the modified Kirby-Bauer diffusion method. Gram-negative (*Escherichia coli*; ATCC: 9637 and *Klebsiella pneumonia*; ATCC: 10,031) and Gram-positive (*Streptococcus mutans*; ATCC: 25,175 and *Staphylococcus aureus*; ATCC: 6538) bacterial strains and *Candida albicans* fungi (ATCC:10,231) were used for the test. Briefly, 100 μl of log 8 CFU/g or ml (Colony-Forming Unit) of a pure overnight culture of each strain grown in nutrient broth at 35 °C ± 2 °C and shaken at 150 rpm was spread over the surface of well-dried nutrient agar plates. Then, wells of 6 mm diameter were inoculated individually with 100 μl (15 mg/ml) of the tested modified nanopigments dissolved in dimethyl sulfoxide (DMSO). Ampicillin, gentamicin and nystatin were used as a positive control for Gram-negative, Gram-positive bacterial strains and fungi, respectively. DMSO was used as a negative control. All plates were incubated at 35 °C ± 2 °C for 24 h and observed for clear zone formation. This experiment was performed in triplicate, and zones of inhibition were measured in millimetres.

### Application of the prepared nanopigments in paper coating

#### Preparation of paper coating mixtures

The prepared pigments were dispersed in distilled water with a dispersing agent of 0.3 parts sodium hexametaphosphate at a solid content of 25%. The binder (15 pph) was carefully added to the pigment slurry (pph = part per hundred parts of dry pigment); the agitator was set to a moderate speed to prevent foam formation during the binder addition. Finally, the solid content was adjusted to 50% using water, and sodium hydroxide was added to adjust the pH of the suspensions to 8.

#### Preparation of coated paper samples

The coating combinations were applied using a K-bar semiautomatic coater (R&K print coat instruments Ltd, United Kingdom, model NOS k101)). To make a 6-m-thick wet film, a wire-wound bar was chosen. Precoat white paper samples with a grammage of 90 g/m^2^ were cut to 200 mm × 300 mm using a strip cutter and were coated according to the standard temperature and humidity conditions of 23 ± 1 °C and 50 ± 2% ISO 187.

#### Characterization of coated paper samples

The coated paper samples including the prepared pigments were characterized using standard tests for optical, mechanical and physical properties. Brightness is related to the paper’s overall reflectivity, or visual efficiency. The tests were performed using a brightness and colorimeter device (68–59-00–002, Buchel-B. V, Netherlands) reporting ISO 2470–1 (2009). The same device was used to estimate opacity following ISO 2471 (2008). Burst strength is the hydrostatic pressure in KPa needed to rupture a paper sample in an approximate sphere of 1.2 inches in diameter when deformed at a controlled rate of loading. The measurements were conducted on burst tester, model BT-10 TlS Techlab Systems, according to ISO 2758–3 (2009). Tensile strength in KN/m is the amount of force required to break a narrow strip of paper when the strip’s length and rate of loading are both carefully defined. Tensile test machine, T-series, model H5KT, Tinius Olsen Ltd, at 1 KN was employed according to ISO 1924–2 standard.

#### ***Accelerated ageing of the prepared coated paper samples***

A reliable accelerated ageing method would be desirable for determining paper permanence and biological deterioration since natural ageing requires long periods to generate meaningful trends in paper properties. Coated paper samples were subjected to accelerated ageing at 100 °C for 14, 29, 43 and 72 h in circulating air to obtain 5, 10, 15 and 25 years of natural ageing. They were conditioned prior to tests according to recommendations given in TAPPI Method T 402. This accelerated ageing method aimed to study the ability of Ag@TiO_2_-coated paper to preserve its bacterial character over a long period reaching up to 25 years.

#### Antimicrobial activity of accelerated ageing coated paper

Colony forming unit (CFU) assay was used to test the antimicrobial activity of previously aged, coated papers. *Staphylococcus aureus* (*S. aureus*) was utilized for the test. To evaluate the antimicrobial ratio of the coated paper, *S. aureus* suspension (McFarland standard 0.5) was prepared and incubated in Mueller–Hinton broth medium. Then, 200 μl of the resulting bacterial suspension was then added to 96-well plate containing the aged coated paper samples and control sample (DMSO). After a 24-h incubation period at 37 °C, the number of CFUs was counted. (N/N0) × 100 was used to calculate the antimicrobial efficiency, where *N*_0_ and *N* are the average numbers of acquired CFUs for both control substrate and aged coated sheets, respectively. A digital camera was used to count the number of colonies formed by the microorganisms on the plates (Xiang et al. 2018).

## Results and discussion

### *Characteristics of the as-synthesized Ag@TiO*_*2*_*-modified nanopigments*

**Fig. 1:**
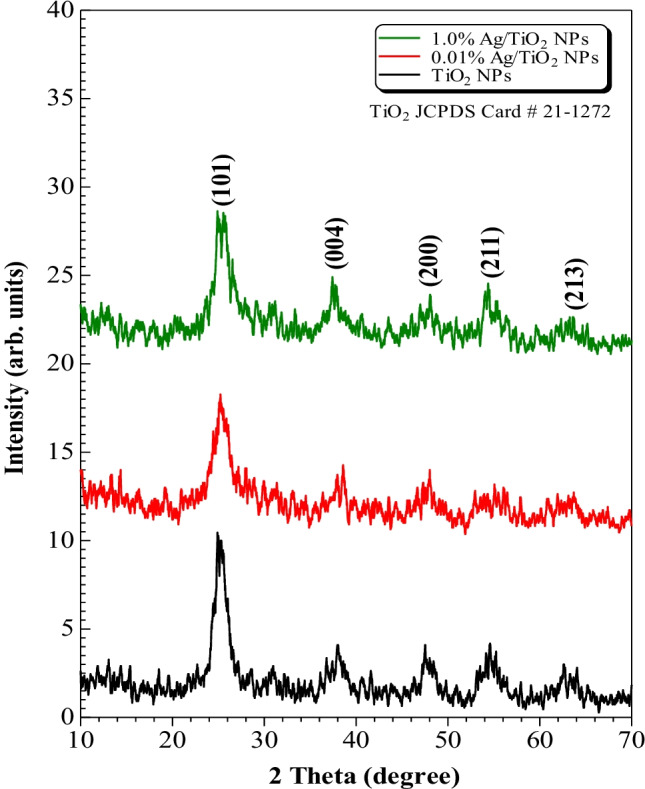
XRD patterns of the as-synthesized TiO_2_ and Ag@TiO_2_-modified nanopigments

The crystal structure of the as-synthesized samples was analyzed with X-ray diffraction instrument. Figure 1 demonstrates the XRD patterns of TiO_2_ and Ag@TiO_2_ samples with various Ag weight ratios. In general, all patterns have nearly broad and weak intensity peaks. This might be due to the nanosize regime of the obtained particles as will be shown later. It is widely known that the widening and low intensity of all sample peaks indicate that the as-synthesized particles have small crystallite sizes (El-Sherbiny et al. 2015; Morsy et al. 2014). All diffraction peaks are consistent with the (101), (004), (200), (211) and (213) crystal planes of TiO_2_ anatase phase (JCPDS card no. 21–1272). Obviously, there is no noticeable difference between TiO_2_ and Ag@TiO_2_ patterns. This might be due to the low content/crystallinity of Ag NPs in the prepared samples.

**Fig. 2:**
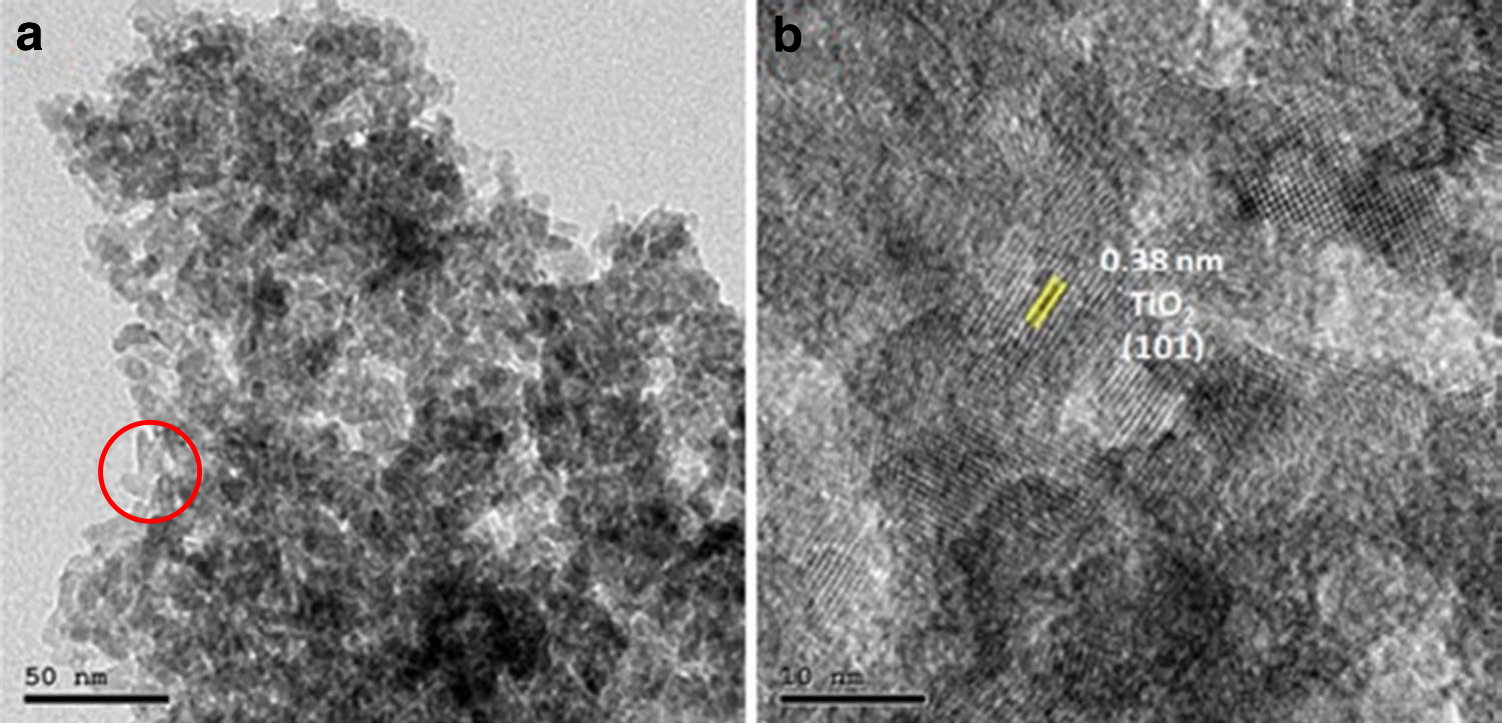
TEM and HRETM images of 0.01% Ag@TiO_2_-modified nanopigments. The dashed line circle (**a**) refers to the formed nanorod and the two solid lines (**b**) refer to the lattice fringe

The TEM and HRTEM images of the as-synthesized 0.01% Ag@ TiO_2_ are presented in Fig. 2. The results indicated that the formed TiO_2_ NPs decorated with Ag NPs are mainly in the form of nanorods with high crystal quality and an average particle size of about 20 nm (Fig. 2a), while the Ag NPs are in the form of quasi spherical particles with average size of about 5 nm according to the HRTEM image (Fig. 2b).

**Fig. 3:**
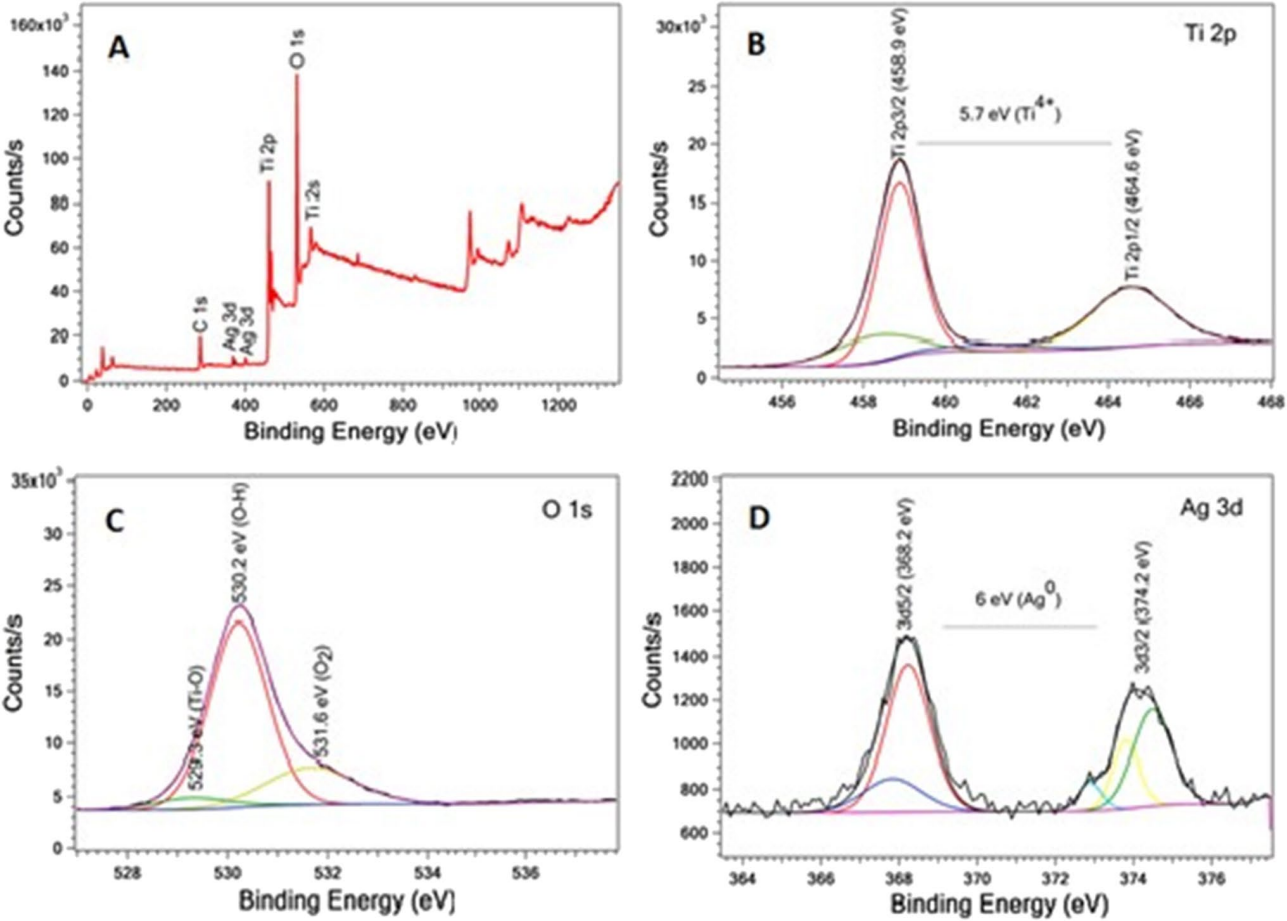
XPS spectrum of 0.01% Ag@TiO_2_ modified nanopigments: **A** survey scan, **B** Ti 2p, **C** O 1 s and **D** Ag 3d narrow scans

To investigate the chemical composition and the oxidation states for the as-synthesized 0.01% Ag@TiO_2_ sample, XPS was performed as shown in Fig. 3. The survey scan of the as-synthesized 0.01% Ag@TiO_2_ sample (Fig. 3A) clearly shows the Ti 2p, O 1 s, C 1 s and Ag 3d XPS peaks. The narrow scan of the aforementioned peaks is represented in Fig. 3B–D. Figure 3B shows that Ti2p XPS peak shows two peaks at binding energy values of 458.9 eV and 464.6 eV which are ascribed to Ti 2p3/2 and Ti 2p1/2, respectively (Fouad et al. 2011). The energy distance between the two peaks is 5.7 eV, which is characteristic of Ti^4+^ oxidation state of TiO_2_ compound (Hariharan et al. 2020). Moreover, the O1s XPS peak (Fig. 3C) is fitted into three peaks. The peak at 529.3 eV could be assigned to the oxygen in Ti–O compounds. While the peak at 530.2 eV reveals that the surface is covered with OH group, the peak at a binding energy of 531.6 eV is most likely related to the adsorbed oxygen. The Ag 3d XPS spectra peak shows two peaks at binding energy values of 368.2 eV and 374.2 eV, which are corresponding to Ag 3d5/2 and Ag 3d3/2, respectively. The energy difference between the two peaks is approximately 6 eV which is assigned to the presence of Ag^0^ oxidation state (Zhang et al. 2018).

**Fig. 4:**
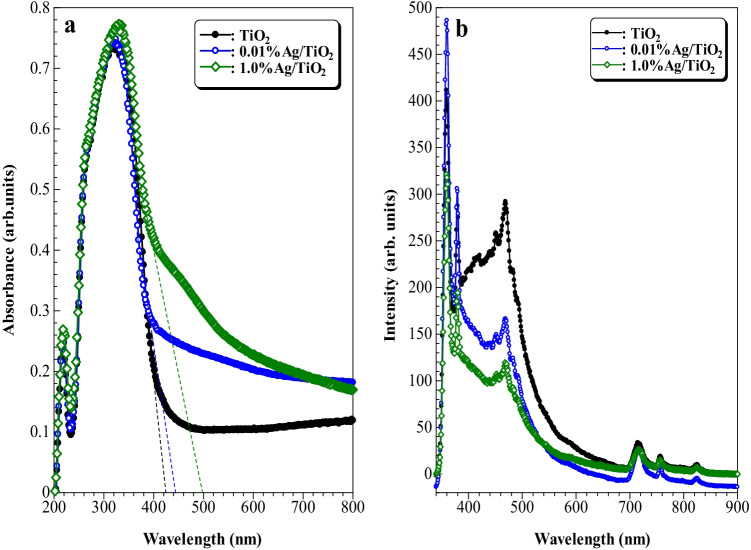
**a** UV–visible absorbance spectra and **b** PL spectra at excitation wavelength of 320 nm of the as-prepared TiO_2_ and Ag@TiO_2_ NPs

The optical properties of the as-prepared Ag@TiO_2_ nanoparticles were evaluated using a diffuse reflectance UV–Vis spectrophotometer and PL as shown in Fig. 4. Obviously, there is a difference between the absorption spectra of pure TiO_2_ and Ag@TiO_2_ NPs (Fig. 4a). By loading Ag NPs, the absorption spectra extended to the visible region. The 1%Ag@TiO_2_ NPs show a broad absorption spectrum in the range of 200–550 nm, implying its photocatalytic activity under visible light. The semiconductor forbidden band width was determined by the equation *Eg* (eV) = 1240/λ_g_ (absorption threshold, λ_g_ (nm)). Accordingly, the band gap of TiO_2_, 0.01%Ag@TiO_2_ and 1%Ag@TiO_2_ NPs was 2.93, 2.82 and 2.49 eV, respectively. Figure 4b shows the PL spectra of the pure TiO_2_ and Ad@TiO_2_ NPs with an excitation wavelength of 320 nm. It is clearly seen that all samples have the same emission peaks. However, the PL intensity of Ag@TiO_2_ samples is lower than that of pure TiO_2_. Therefore, Ag@TiO_2_ NPs performed a good electron–hole separation and consequently enhancing the photocatalytic activity.

### *Antimicrobial activity tests of the prepared Ag@TiO*_*2*_* NPs*

Table 1 shows the results of the agar well diffusion test for TiO_2_, 0.01% Ag@TiO_2_ and 1% Ag@TiO_2_ modified nanopigments. The agar well diffusion test is a semi-quantitative method for determining the ability of antimicrobial materials to impede microorganisms’ growth. An inhibition zone forms around the sample during incubation if the antimicrobial substance can spread from the sample into the nutrient agar. The concentration and rate of diffusion of antimicrobial agents from the sample into the agar influence the size of the zone of inhibition (Albert et al. 2015). The recorded values in Table 1 are the average of three readings. The results reveal that TiO_2_ nanoparticles showed various degrees of antimicrobial effect towards the selected Gram-positive, Gram-negative and fungi strains, and the inhibition zones vary from 10.3 to 17.6 mm. Doping TiO_2_ nanopigments with 0.01 Ag nanoparticles shows strong antimicrobial activity with inhibition zone ranging from 11.6 to 17.9 mm compared with pure TiO_2_ nanopigment. Increasing the Ag nanoparticle concentration in the prepared modified nanopigments led to a significant improvement in the antimicrobial properties. For Gram-negative bacteria (*Escherichia coli*, *Klebsiella pneumonia* and *Pseudomonas aeruginosa*), the inhibition zones were 16.6, 16.3 and 10.6 with percentages increase 43.1, 29.4 and 18.9%, respectively. For Gram-positive bacteria (*Staphylococcus aureus* and *Streptococcus mutans*), the inhibition zones reached 22.3 and 15.3 with an improvement of 26.7 and 48.5%, respectively.

**Table 1:** Antimicrobial activity tests of the prepared modified nanopigments

Sample and microorganism	TiO_2_	0.01% Ag@TiO_2_	1% Ag@TiO_2_	Standard antibiotic
Gram-negative bacteria				Gentamicin
* Escherichia coli (ATCC:10,536)*	11.6 ± 0.5	14.6 ± 0.5	16.6 ± 0.6	27 ± 0.5
* Klebsiella pneumonia (ATCC:10,031)*	12.6 ± 0.5	12.3 ± 0.5	16.3 ± 0.6	25 ± 0.5
* Pseudomonas aeruginosa (ATCC:27,853)*	10.6 ± 0.5	11.6 ± 0.5	12.6 ± 0.5	30 ± 0.5
Gram-positive bacteria				Ampicillin
* Staphylococcus aureus (ATCC:13,565)*	17.6 ± 0.6	17.9 ± 0.6	22.3 ± 0.5	22 ± 0.1
* Streptococcus mutans (ATCC:25,175)*	10.3 ± 0.5	11.6 ± 0.5	15.3 ± 0.5	30 ± 0.5
Fungi				Nystatin
* Candida albicans (ATCC:10,231)*	11.6 ± 0.5	12.3 ± 0.5	18.6 ± 0.6	21 ± 0.5

Zone of inhibition is expressed in the form of mean ± standard deviation (mm)

For fungi strain, the prepared modified nanopigments (0.01% Ag@TiO_2_ and 1% Ag@TiO_2_) have a significant effect as antimicrobial upon increasing the Ag concentration; the inhibition zones were 12.3 and 18.6 mm with an improvement of 14.7 and 60.4%, respectively.

The mechanism of antimicrobial properties metal oxides semiconductors, including TiO_2_, is well established, which is related to the generation of ROS (reactive oxygen species), particularly hydroxyl free radicals and peroxide, when exposed to UV-A irradiation (300 nm < l < 390 nm) as it is commonly used as a photocatalyst. To enhance its photocatalytic activity in visible light, two possible routes have been employed: reducing its size to the nanoscale regime and/or doping by metals or non-metals. Through these routes, the energy gap could be controlled, and its photocatalytic properties could be improved (Fouad et al. 2006; Mahmoud and Fouad 2015). The antimicrobial activity of Ag NPs is based on several suggested mechanisms, among these, the damage of the cell wall which leads to an increase in the permeability and leakage of intracellular substances. Additionally, alteration in envelope proteins and heat shock proteins, dissipation of the proton motive force, the collapse of the plasma membrane potential and decrease of intracellular can be also possible antimicrobial mechanisms. Moreover, the liberation of silver ions increases the bactericidal effect through generation of reactive oxygen species (ROS), interacting with thiol groups of the proteins and loss of bacterial replication via DNA condensation leading. Ag nanoparticles also have been proven to be effective antimicrobial agents against both Gram − and Gram + bacteria (Chand et al. 2020; Hileuskaya et al. 2020; Waldo-Mendoza et al. 2016).

### Optical properties

**Fig. 5:**
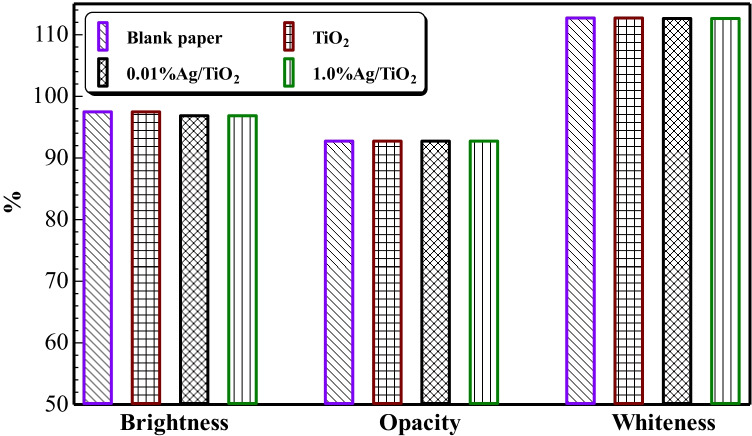
Optical properties of the prepared coated paper

The perceived value of paper products is determined not only by their functionality but also by their aesthetic application. The visual perception and appeal of paper are influenced by its optical qualities, such as whiteness, brightness and opacity. The growing demand for imparting new function properties as antimicrobial activity and strength must does not negatively affect the paper optical quality. To address these challenges, the effect of increasing Ag content in Ag@TiO_2_-modified nanopigments on the optical properties and appearance of coated paper was studied. It is necessary to know how processing and variations in the composition of paper coating mixture will affect the optical properties. Figure 5 shows the obtained results of the optical properties of the prepared coated paper. The results showed that increasing the content of Ag to 1% in Ag@TiO_2_-modified nanopigments has no significant effect on the coated paper’s optical properties compared with high-quality blank paper and pure TiO_2_-coated paper despite the Ag NPs’ grey black colour. These superior properties can be linked to the small content of Ag-doped coated paper and its good distribution in the TiO_2_ matrix porous system.

### Mechanical properties

The paper-coated mechanical properties are vital in the investigation of the bending and compressive deformation that coated paper faces in a printing machine, e.g. paper handling and runnability (El-Sherbiny and Ahmed 2018). The tensile strength and tensile stretching of the TiO_2_-coated paper without Ag NP doping are 6.03 kN/m and 1.55%, respectively.

**Fig. 6:**
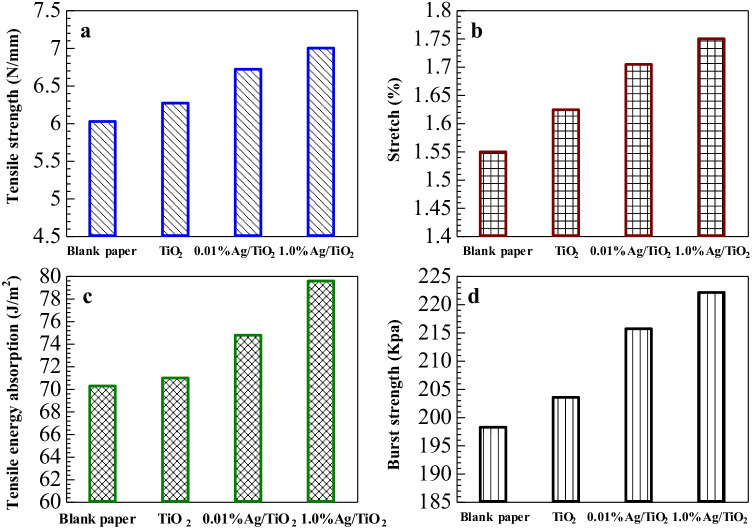
Mechanical properties of the prepared coated **a** tensile strength, **b** stretch, **c** tensile energy absorption and **d** burst strength

Tensile strength increased significantly as the Ag content in the Ag@TiO_2_ coating layer increased when compared to TiO_2_-coated paper. As shown in Fig. 6a, when the content of Ag increased to 1%, the tensile strength increased to the maximum value of 7.00 kN/m, and the degree of improvement is up to nearly 16.08%. At the same time, the tensile stretch increased to 1.75%, and the degree of improvement is about 12.9% as shown in Fig. 6b. Tensile energy absorption (TEA) is a measure of paper’s ability to absorb energy under variable loading conditions and can be used to measure paper durability when repeated straining is required. Figure 6c shows that TEA of the TiO_2_-coated paper increased from 70.03 to about 79.6 J/m^2^ with an improvement of nearly 13.66% using 1% Ag@TiO_2_ nanopigment. The burst strengths of coated papers showed a similar variation tendency; they increased with increasing Ag NP contents. Figure 6d shows that the burst strength of paper coated with 1% Ag@TiO_2_ modified nanopigment increased with a percentage of 12.04% compared with pure TiO_2_-coated paper. It is worth mentioning that the obtained results in Figs. 5 and 6 are the average of 10 readings according to the standards mentioned above in “Characterization of coated paper samples.”

These results are linked to the fact that when metal cations are added to the anatase phase, with a valence band less than + 3, or larger radii than Ti^4+^ ions occupy substitutional positions in the TiO_2_ matrix (Mosquera et al. 2016). Furthermore, the presence of nanoparticles in TiO_2_ hybrid matrix composites prevents dislocation motion and grain boundary migration, and that will cause enhancement of the nanocomposite strength (Annaz et al. 2020; Irhayyim et al. 2019). The strength enhancement can be regarded by increasing the nanosized silver content to the semi-homogeneous distribution of Ag nanoparticles in TiO_2_ matrix and the structure of the formed Ag@TiO_2_-modified nanopigments. It should be mentioned that the coating formulation has an impact on mechanical property improvement.

### Antimicrobial activity tests for the aged coated paper samples

**Fig. 7:**
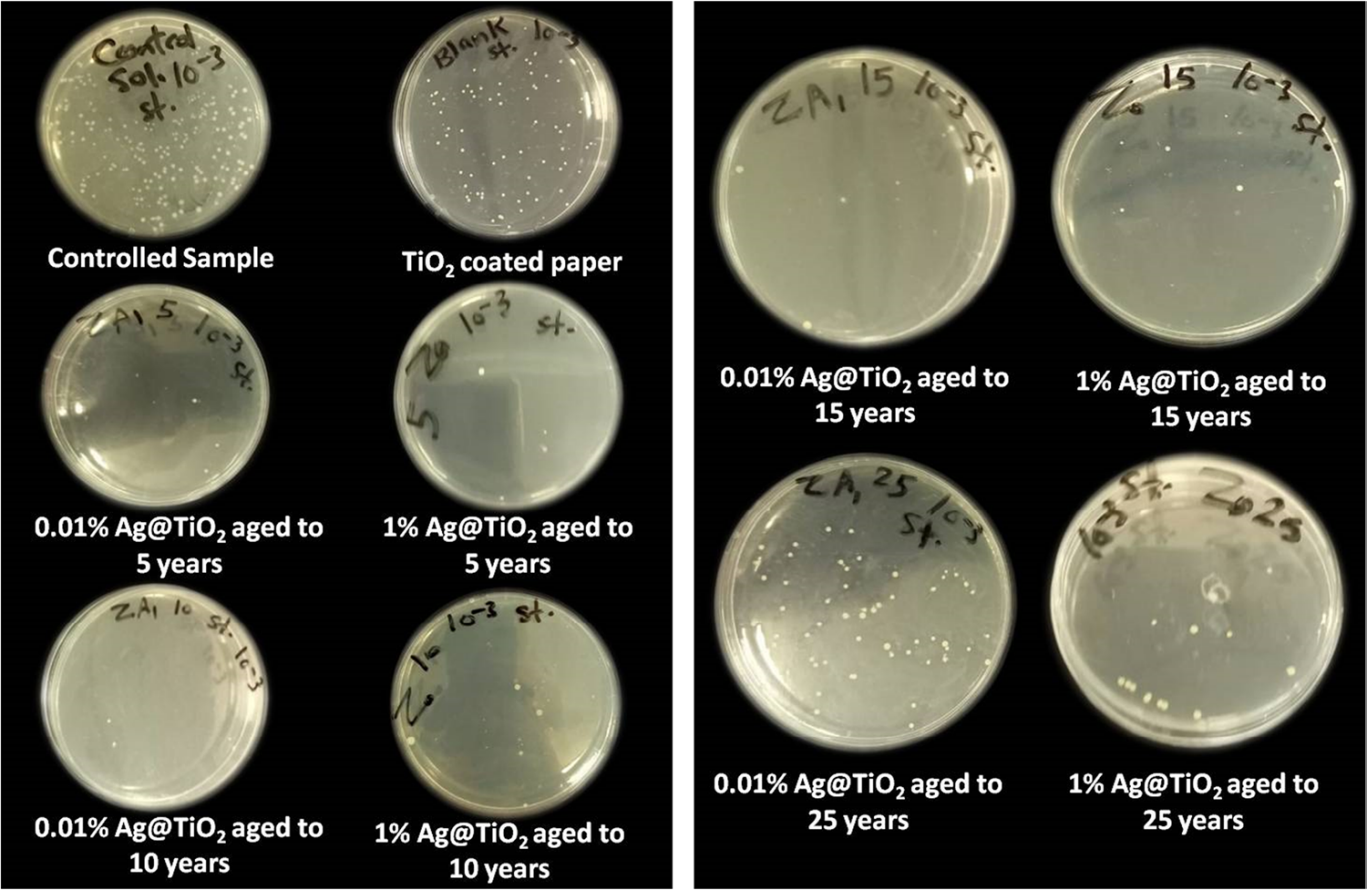
Antimicrobial Activity for the Prepared aged coated paper samples

The paper without antimicrobial properties is easy to be attacked by bacteria in a humid environment. This will seriously affect its service life especially if this exposure was for a long time. As was mentioned above, to improve and enhance the antimicrobial activity of the TiO_2_ surface coating layer, Ag NPs were introduced with the hope of imparting excellent antimicrobial properties to the surface of the paper. Table 2 and Fig. 7 show the obtained results of the CFU assay antimicrobial tests of the long-term aged-coated papers. The CFU assay is a quantitative method that shows the viability of bacterial cells after being incubated on the surfaces. They give the results of the number of colony-forming units (CFU), for the number of grams or millilitres of test material that they put where a colony of microbes grow on the petri dish. The recorded values are the results of the average of three readings. Although the coatings without doping silver show that antimicrobial inhibition reached 59.4%, the inhibition effect increases, and the colony-forming unit decreases significantly by increasing the incorporation per cent of silver into Ag@TiO_2_ coatings. Comparing the antimicrobial effect of the aged-coated paper after a long period of time (25 years), coated paper with 1% Ag@TiO2 showed 93.4% inhibition efficiency compared with 70.6% for the 0.01% Ag@TiO2. Samples with higher Ag content remain active against bacteria even after 25 years. These advantageous properties can be attributed to the nature of the Ag NPs where they impregnate into the porous structure due to the size-limiting character of the mesoporous paper surface. This makes it an ideal candidate for the application in coating paper–based materials.

**Table 2:** Antimicrobial activity of aged coated paper samples

*Staphylococcus aureus*	TiO_2_-coated paper	0.01% Ag@TiO_2_				1% Ag@TiO_2_				Control
		5Y	10Y	15Y	25Y	5Y	10Y	15Y	25Y	
Dilution factor	10.^−3^	10.^−3^	10.^−3^	10.^−3^	10.^−3^	10.^−3^	10.^−3^	10.^−3^	10.^−3^	10.^−3^
Volume of broth plated (µl)	20 µl	20 µl	20 µl	20 µl	20 µl	20 µl	20 µl	20 µl	20 µl	20 µl
Colony forming unit (CFU) at the dilution factor	80	3	4	8	58	2	5	7	13	211
Total CFU	3,000,000	112,500	150,000	300,000	2,175,000	75,000	187,500	262,500	487,500	7,387,500
Log total CFU	6.48	5.05	5.18	5.48	6.34	4.88	5.27	5.42	5.69	6.87
Per cent of inhibition	59.4%	98.5%	98%	95.9%	70.6%	99%	97.5%	96.5%	93.4%

### Possible mechanism for antibacterial activity

It is well known that TiO_2_ under UV irradiation generates reactive oxygen species (ROS) such as ·OH, O_2_^−^ and H_2_O_2_ (Chokesawatanakit et al. 2021). The ROS degrades peptidoglycan in the cell wall and interacts with sulfhydryl groups of proteins and DNA, causing damage to proteins and DNA as follows:

1 $${\mathrm{TiO}}_{2}+\mathrm{hv}\to {\mathrm{TiO}}_{2}\left({{\mathrm{e}}^{-}}_{\mathrm{CB}}+{{\mathrm{h}}^{+}}_{\mathrm{VB}}\right)$$

2 $${\mathrm{TiO}}_2\left({\mathrm h^+}_{\mathrm{VB}}\right)+{\mathrm H}_2\mathrm O\rightarrow\cdot\mathrm{OH}+\mathrm H^++{\mathrm{TiO}}_2$$

3 $${\mathrm{TiO}}_2\left({\mathrm e^-}_{\mathrm{CB}}\right)+{\mathrm O}_2\rightarrow{\cdot{\mathrm O}_2}^-$$

4 $$\mathrm H^++{\mathrm O}_2^-\rightarrow\cdot\mathrm{OOH}$$

5 $${\mathrm e^-}_{\mathrm{CB}}+\mathrm H^++\cdot\mathrm{OOH}\rightarrow{\mathrm H}_2{\mathrm O}_2+2\mathrm{OH}$$

6 $${\mathrm{TiO}}_2\left({\mathrm h^+}_{\mathrm{VB}}\right)+\mathrm{cellwall}\rightarrow\mathrm{free}\;\mathrm{radical}\;\mathrm{on}\;\mathrm{peptidoglycan}\;\mathrm{in}\;\mathrm{cell}\;\mathrm{wall}+\mathrm{Ti}{\mathrm O}_2$$

**Scheme 1:**
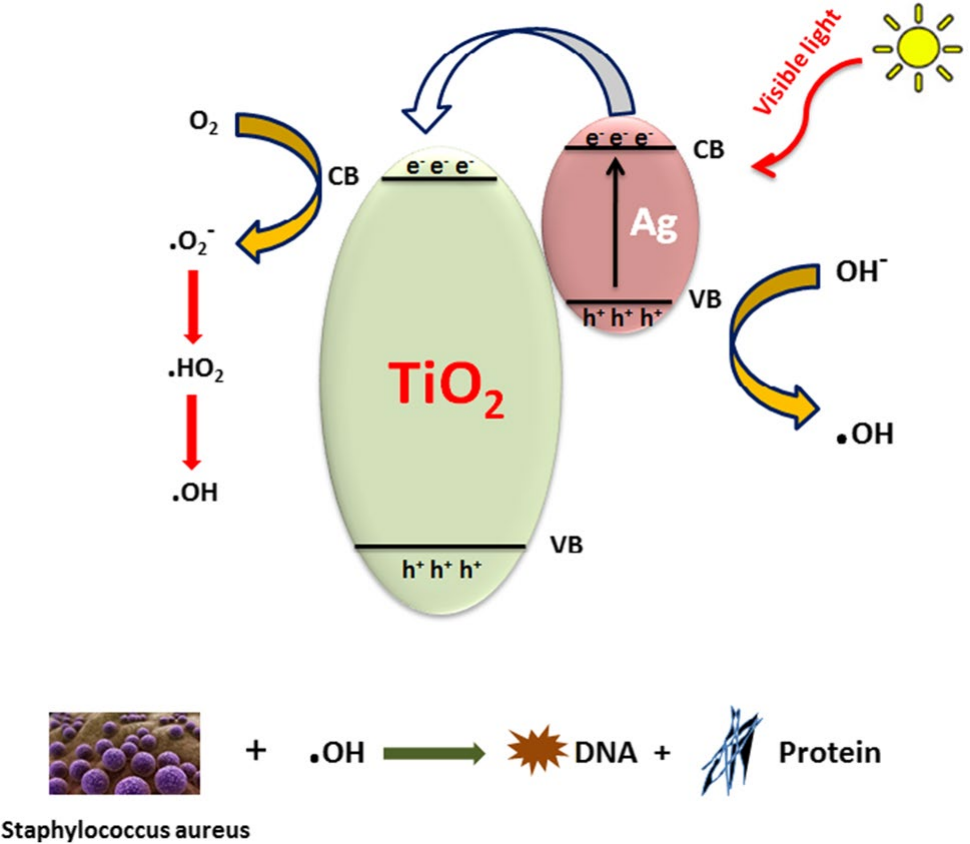
Schematic diagram of visible light–induced antibacterial mechanism of Ag/TiO_2_ NPs

In contrast, Ag/TiO_2_ under visible light irradiation possesses a different scenario. The generated electrons are transferred from the CB of Ag nanoparticles to the CB of TiO_2_ (Eq. 8). As the conduction band of titanium dioxide is an electron acceptor, it readily accepts the electrons and forms superoxide anion radicals and followed by the protonation that yields hydroxyl radicals and hydroxide ions (Eq. 9 and 10). The holes at Ag NPs react with H_2_O and OH^−^ to form hydroxyl radicals (Eqs. 11 and 12). The effective electron–hole pair’s separation results in a higher generation of OH radicals, implying enhancement of the catalytic activity. Furthermore, the OH radicals will penetrate the cell causing degradation of cell wall and cytoplasmic membrane of the bacteria (Khan et al. 2016; Mansor et al. 2021; Surendra and Roopan 2016) as proposed in Scheme 1.

7 $$\mathrm{Ag}+\mathrm{visible light}\to \mathrm{Ag}\left({\mathrm{e}}^{-}\right)+\mathrm{Ag}\left({\mathrm{h}}^{+}\right)$$

8 $$\mathrm{Ag }\left(\mathrm{e}^-\right)+{\mathrm{TiO}}_{2}\to {\mathrm{TiO}}_{2}\left({\mathrm{e}}^{-}\right)$$

9 $${\mathrm{TiO}}_2\left(\mathrm e^-\right)+{\mathrm O}_2\rightarrow\cdot{\mathrm O}_2^-$$

10 $$\cdot{\mathrm O}_2^-+\mathrm e^-+\mathrm H^+\rightarrow\cdot\mathrm{OH}+\mathrm{OH}^-$$

11 $$\mathrm{Ag}\left(\mathrm h^+\right)+{\mathrm H}_2\mathrm O\rightarrow\cdot\mathrm{OH}$$

12 $$\mathrm{Ag}\left(\mathrm h^+\right)+\mathrm{OH}^-\rightarrow\cdot\mathrm{OH}$$

## Conclusion

A new approach for imparting dual function properties in paper coating was presented. The modification involved the preparation of Ag@TiO_2_ nanopigments for surface paper coating via a simple and feasible technique. Ag-doped anatase phase with nanorod-like structure of about 20-nm average size was obtained and confirmed by XRD and TEM image analyses, respectively. The effect of the amount of the silver nanoparticle dopant of the composite coatings was studied in terms of paper coating properties and antimicrobial activities. Pure TiO_2_ nanoparticle showed a good antimicrobial effect, and increasing the content of Ag NPs doping significantly improved its performance against six strains (Gram-negative, Gram-positive and fungi). Coated papers with Ag@TiO_2_-modified nanopigments remain active against *Staphylococcus aureus* bacteria even after ageing for 25 years. Coated paper with Ag@TiO_2_ with a per cent of 1% reached 93.4% bacterial inhibition compared to 70.6% with 0.01% Ag@TiO_2_. Ag@TiO_2_-modified nanopigments endowed the coating with higher mechanical properties with no significant change in optical properties despite Ag grey dark colour. Making equilibrium between enhancement of paper mechanical properties and antimicrobial activity without decreasing in optical properties is the main demand to achieve superior paper quality. Increasing Ag NP content to 1% improved tensile strength, stretch, tensile energy absorption and burst strength of coated paper with 16.3, 12.9, 13.22 and 12.04%, respectively. Hence, applying Ag@TiO_2_-modified nanopigment paper coatings is a good candidate for enhancing paper properties which indicated that coated paper had superior mechanical stress and bacterial infection inhibition to cope with handling, humid environments and runnability.

## Data Availability

The original data that support the findings of this study are available upon request.
